# Compound C Reducing Interferon Expression by Inhibiting cGAMP Accumulation

**DOI:** 10.3389/fphar.2020.00088

**Published:** 2020-02-28

**Authors:** Junzhong Lai, Xuan Luo, Shuoran Tian, Xing Zhang, Shanlu Huang, Hanze Wang, Qiumei Li, Shaoli Cai, Qi Chen

**Affiliations:** ^1^ Fujian Key Laboratory of Innate Immune Biology, Biomedical Research Center of South China, Fujian Normal University Qishan Campus, Fuzhou, China; ^2^ College of Life Science, Fujian Normal University Qishan Campus, Fuzhou, China

**Keywords:** compound C, dorsomorphin, cGAS, cGAMP, DNA sensing, type I interferon

## Abstract

Cyclic GMP-AMP (cGAMP) synthase (cGAS) is a major DNA sensor responsible for cytosolic DNA-mediated innate immune response. Inhibition of cGAS may be an effective strategy for treating autoimmune diseases such as Aicardi-Goutieres syndrome and systemic lupus erythematosus. Compound C (also known as Dorsomorphin) has been annotated as a potent and reversible inhibitor for AMPKs as well as ALK protein kinases. Here, we report a new function of Compound C which can suppress dsDNA-dependent type I interferon induction. These effects were not dependent on the activities of AMPK proteins. *In vitr*o assays and liquid chromatograph-mass spectrometry data show that Compound C has the capability of reducing cGAMP accumulation, suggesting that Compound C may function as a modulator involved in the cGAS-STING-mediated DNA sensing pathway. Furthermore, Compound C is able to rescue the autoimmune phenotypes in a mouse model carrying the Trex1 gene deficiency. These data demonstrate a new and inverse correlation between Compound C and type I interferon production in response to dsDNA signaling.

## Introduction

Innate immune response represents a fundamental ability of host-defense in dealing with pathogen invasion or endogenous tissue injury (Takeuchi and Akira, 2010). DNA, as a dangerous immune stimulant, can be derived from a large variety of sources, including invading microbes and self-destructive cells, and trigger host innate immune response (O'Neill et al., 2013). Recognition, clearance, and signaling of these dangerous DNAs play a pivotal role in host defense and human health (Goubau et al., 2013; Wu and Chen, 2014; Pandey et al., 2015). Cyclic GMP-AMP (cGAMP) synthase (cGAS) has been discovered as a major DNA sensor that recognizes the dangerous DNAs present in the cytoplasm and is responsible for cytosolic DNA-mediated immune responses leading to the production of type-I interferons (IFNs) and other inflammatory cytokines. cGAS binds B-form DNA in a sequence-independent manner (Xiao and Fitzgerald, 2013; Cai et al., 2014; Wu and Chen, 2014), and is considered to contribute as a nonredundant and dominant cytosolic DNA sensor (Li et al., 2013; Schoggins et al., 2014). DNA binding causes the activation of cGAS, catalyzing the synthesis of non-canonical 2′-5′ cGAMP (Ablasser et al., 2013; Civril et al., 2013; Diner et al., 2013; Gao et al., 2013; Kranzusch et al., 2013; Li et al., 2013; Zhang et al., 2013; Abe and Barber, 2014; Zhang et al., 2014). Then, cGAMP acting as a second messenger to stimulate the adaptor protein STING, along with STING rapidly traffics with TBK1 *via* VPS34-related autophagosomes to associate with endosomal compartments containing NF-κB and IRF3 (Ishikawa and Barber, 2009; Abe et al., 2013), and triggers the production of numerous cytokines and chemokines responsible for innate immune response.

Due to the significant role of cGAS in innate immunity, small-molecule inhibitors of cGAS may be used not only for further exploring cGAS-mediated DNA sensing mechanisms and innate immunity regulation, but also for treatments of autoimmune disorders (Vincent et al., 2017). Recently, small molecules such as RU.521 and RU.365 have been found to bind to the catalytic pocket of cGAS and inhibit its dsDNA-stimulated activity. Unfortunately, RU.521 only showed the effect in a cellular assay, but not in the *in vivo* tests (Vincent et al., 2017). PF-06928215, a high affinity inhibitor of human cGAS activity (IC50 = 4.9 μM), displayed no activity in cellular cGAS assays when measuring dsDNA-induced IFN-β expression (Hall et al., 2017). In addition, Suramin has been identified as a new cGAS inhibitor, but its activity needs to be further validated (Wang et al., 2018b). Thus, it is a daunting challenge to discover the better cGAS inhibitors both *in vivo* and *in vitro*.

The previous publication demonstrated that STING is activated by UNC-51-like kinase (ULK1)-mediated phosphorylation, which occurs following ULK1 dissociation from its repressor, AMP-activated protein kinase (AMPK), and inhibition of AMPK by compound C substantiated ULK1-mediated STING phosphorylation and activation (Konno et al., 2013).

Compound C is a small molecule compound commonly used as an inhibitor of AMPK which is the key energy sensor in cells (Zhou et al., 2001; Jin et al., 2009). However, Compound C has been shown to exert various AMPK-independent effects in different cell types (Seo et al., 2016). For example, treatment of MCF7 cells by Compound C leads to Bax redistribution from the cytoplasm to mitochondria and cell death (Jin et al., 2009), and Compound C increases Sestrin2 expression *via* mitochondria-dependent ROS production (Seo et al., 2016). Compound C prevents the AMPK signaling-independent unfolded protein response during glucose deprivation (Saito et al., 2012). Compound C inhibits ICAM-1 and VCAM-1 expression in inflammatory stimulants-activated endothelial cells (Kim et al., 2011). In accordance with these discoveries, Compound C has been found to inhibit many other kinases in addition to AMPK in several kinase profiling studies and is thus highly non-specific (Dasgupta and Seibel, 2018).

In this study, the important role of Compound C related to innate immunity was investigated. We found that Compound C could largely inhibit type I interferon production induced by foreign DNAs, but not by cGAMP. Compound C mediated DNA-induced IFN inhibition might occur in the upstream of cGAMP and reveal a new functional role of Compound C in addition to its existing inhibitory activities in many kinases-involved signaling pathways.

## Materials and Methods

### Cell Culture and Transfection

L929, BJ, THP1, and 293T cell were cultured in an atmosphere of 5% CO_2_ in RPMI-1640 or DMEM medium supplemented with 10% fetal bovine serum (FBS). THP1-lucia-IFNβ-ISG was purchased from Invivogen (California, USA) and cultured in an atmosphere of 5% CO_2_ in RPMI-1640 medium supplemented with 10% FBS after 55°C inactivated.

Transfection of HT-DNA (Sigma, St. Louis, Missouri, USA) and plasmid DNA (pcDNA-3.1-TBK1-Flag) into cells were performed by mixing 2 μg DNA with 6 μl Lipofectamine 2000 (Invitrogen, California, USA). cGAMP (Biolog, Flughafendamm, German) stimulation assay was performed as previously described (Wu et al., 2013). Briefly, cells were incubated at 37°C for 30 min with cGAMP in permeabilization buffer (50 mM HEPES, pH 7; 100 mM KCl; 3 mM MgCl_2_; 0.1 mM DTT; 85 mM sucrose; 0.2% BSA; 1 mM ATP, 0.1 mM GTP and 1μg ml-1 digitonin). Then, the permeabilization buffer was replaced with complete medium and cells were cultured for the indicated time.

### Mouse Embryonic Fibroblast Culture

The Trex1^-/-^ mouse line was obtained from the Jackson Laboratories (Cambridge, MA, USA). All mice were maintained under pathogen-free conditions and housed in a temperature (22°C ± 2°C) and humidity controlled environment on a 12-h light/dark cycle with free access to food and water. The animal experiments were performed under the Guide for the Care and Use of Laboratory Animals approved by Fujian Provincial Office for Managing Laboratory Animals and were overseen by the Fujian Normal University Animal Care and Use Committee.

Primary MEFs were isolated from embryonic day 13.5 (E13.5) embryos of wild type and Trex1^-/-^ mice. MEFs were cultured in the DMEM supplemented with 10% FBS with the addition of 100 U/ml penicillin and 100 mg/ml streptomycin and under the culture condition that includes 37°C with 5% CO_2_.

### Cell Viability Assay

Cell were seeded into 96-well plates at a density of 5 × 10^4^ cells per well and incubated with Compound C (the purity is 99.82% and purchased from Selleck, Shanghai, China) at the indicated concentration for 24 h. The cell viability was analyzed with CCK-8 (TransGen Biotech, Beijing, China) according to the manufacturer’s instruction.

### RNA Isolation, Reverse Transcription (RT), and Real-Time Quantitative Polymerase Chain Reaction (qPCR)

Total RNA was extracted from cells with Trizol (TAKARA, Dalian, China) according to the manufacturer’s instruction. The RT reaction was performed using 1 µg of total RNA with PrimeScript^®^ RT reagent Kit plus gDNA Eraser (TAKARA, Dalian, China).

The mRNA expression levels of different genes were quantified by the real-time quantitative RT PCR (RT-qPCR) using SYBR^®^ Premix Ex Taq II (Tli RNaseH Plus) (TAKARA, Dalian, China) at 95°C for 30 s, followed by 40 cycles of 95°C 5 s, 60°C 20 s, and 72°C 30 s. The PCR was performed on QuantStudio™ 6 Flex Fast real-time PCR system (Applied Biosystems, Carlifornia, USA), and the relative expression level was calculated by using the 2^-△△CT^ method. The primer sequences for related genes are shown in **Table S1** of supplementary materials.

### Generation of Gene Knockout Cell Lines *via* CRISPR/Cas9

The sgRNA oligo sequences for their respective target genes are as follows: m-AMPKα1: 5′-CGAGTTGACCGGACATAAAG; m-AMPKα2: 5′-CCTGAAGCGAGCGACTATCA; H-STING: 5′-GGTGCCTGATAACCTGAGT. The sgRNA sequences were annealed and cloned into the vector PX459. To delete target genes, L929 and THP1 cells were transiently transfected with the PX459 plasmids carrying the respective sgRNAs, and selected with 1 μg/ml puromycin for 2 days. Cells were then cultured in complete medium without puromycin and seeded at a low density to allow colony formation from single cells. Colonies were then picked and expanded for knock-out validation by sequencing of target genomic region, immunoblotting or ELISA.

### Western Blot Analysis and ELISA

The cells with the various treatments were washed with ice-cold PBS, harvested by gentle scraping, and lysed with ice-cold RIPA cell lysis buffer (25 mM Tris•HCl pH 7.6, 150 mM NaCl, 1% NP-40, 1% sodium deoxycholate, 0.1% SDS). Total protein amount was determined by BCA protein determination method. Forty microgram of the protein samples were electrophorased on 10% Tricine-SDS-polyacrylamide gels and transferred to polyvinylidene difluoride membranes for hybridization with the corresponding primary antibodies, followed by IRDye 800CW or 680 LT secondary antibodies (1:1,000) and visualized by Odyssey CLx Western Blot Detection System (Westburg, Leusden, Netherlands). The expression of GAPDH was used as the endogenous control. The level of IFNβ production was measured by ELISA according to manufacturer’s instructions of mouse IFNβ bioluminescent ELISA kit (Invivogen, Carlifornia, USA).

### Preparation of S100 Cytoplasmic Extracts

The BJ cells (1×10^6^ cells per 10 cm^2^ flask) were treated with DMSO or the various concentrations of Compound C (3 μM, 5 μM, 10 μM, 20 μM) for 1 h. Then, the cells were treated with or without HT-DNA for 6 hours and the cytoplasmic extracts (S100) were prepared as follows: the attached cells were firstly soaked in hypotonic buffer (10 mM Tris-HCl, pH7.4, 10 mM KCl, 1.5 mM MgCl_2_). Then, the cells were scraped down and centrifuged at 14,000g for 10 min. The supernatant was collected and heated at 95°C for 5 min. Finally, the cell lysate mixtures were centrifuged at 14,000g for 8 min, and the supernatant was collected, designated as the cytoplasmic extract (S100). The cytosolic extracts from mock or HT-DNA-transfected cells were then permeabilized by incubating with PFO in THP1-Lucifrase cells and the expression of IFNβ was analyzed by RT-qPCR.

### *In Vitro* cGAMP Synthesis Assay

The cGAS enzyme activity detection mixture was prepared as follows: 1 mg/ml HT-DNA, purified recombinant mouse and human cGAS protein (the cGAS expression vector was constructed in our laboratory), 10×buffer (200 mM Tris-Cl, 50 mM MgCl_2_, pH7.5) ,0.1 M CoCl_2_, 100 μM ATP, 100 μM GTP). Then the mixture was aliquoted and incubated with various concentrations of Compound C at 37°C for 30 min. Finally, the ATP of reaction mixture was detected by ATP Assay Kit.

### cGAMP Quantitative Analysis by LC/MS

cGAMP was extracted and purified from BJ cell treated with Compound C, and the quantity of cGAMP was detected by mass spectrometry. Cell extracts were obtained by using 20% (vol/vol) methanol and 2% acetic acid solution after transfected with HT-DNA for 6 hours. cGAMP labeled with 13C1015N5 was added as the homogenized control. Then, cGAMP of the cell extracts was enriched by using the HyperSep Aminopropyl SPE column and the eluent was dried by rotary vacuum and redissolved in liquid chromatography (LC)/MS grade water. Finally, the enriched cGAMP was transferred to the automatic sampler vials of the liquid chromatograph/mass spectrometer for mass spectrometry analysis as follows.

The SPE eluent was separated by Dionex Ultimate 3000 rapid liquid chromatography system (Thermo Scientific, Massachusetts, USA), using Xbirdgy Amide column (3.5 µM, inner diameter 3.5 mm ×100 mm, Waters). The mobile phase A was 20 mM ammonium bicarbonate aqueous solution containing 20 mM ammonium hydroxide, and the mobile phase B was acetonitrile. The first separation velocity was 400 ml/min and the time was 14.5 min. The flow rate was 800 ml/min and the time was 8.5 min. The gradient elution process was: 85% B 0 min, 85% B 3 minutes; 2% B 10 min; 2% B 14 min; 85% B 14.5 min; and 85% B 23 min.

The LC eluent collected by liquid chromatography was ionized by Ion Max NG heating electrospray source, and the spray voltage was 3,750 V. Ion transfer tube temperature was 342°C, gasification temperature was 292°C. The spray was analyzed by TSQ Quantiva triple quadruplet mass spectrometer (Thermo Scientific, Massachusetts, USA), where the continuous multireaction monitoring scanning residence time was 50 ms, the resolution of Q1 and Q3 was 0.7 FWHM, and the collision decomposition gas was 1.5 units. cGAMP and the previously standard cGAMP which was labeled 13C1015N5 were monitored by four transformations respectively in the positive mode (cGAMP: 675-136, 675-152 675-476, and 675-524; Internal standards: 691–146, 691–152, 691–491, and 691–539). The original mass spectrum data would be converted into the mzXML format with ReAdW and read into MATLAB software for noise reduction and data analysis. The absolute amount of cGAMP standard labeled 13C1015N5 was used as a reference to calculate the absolute amount of cGAMP to be tested, so as to obtain the amount of cGAMP contained in the same amount of cells under different conditions.

## Results

### Compound C Inhibits Type I IFN Production Induced by dsDNA Signaling

**Figure 1:**
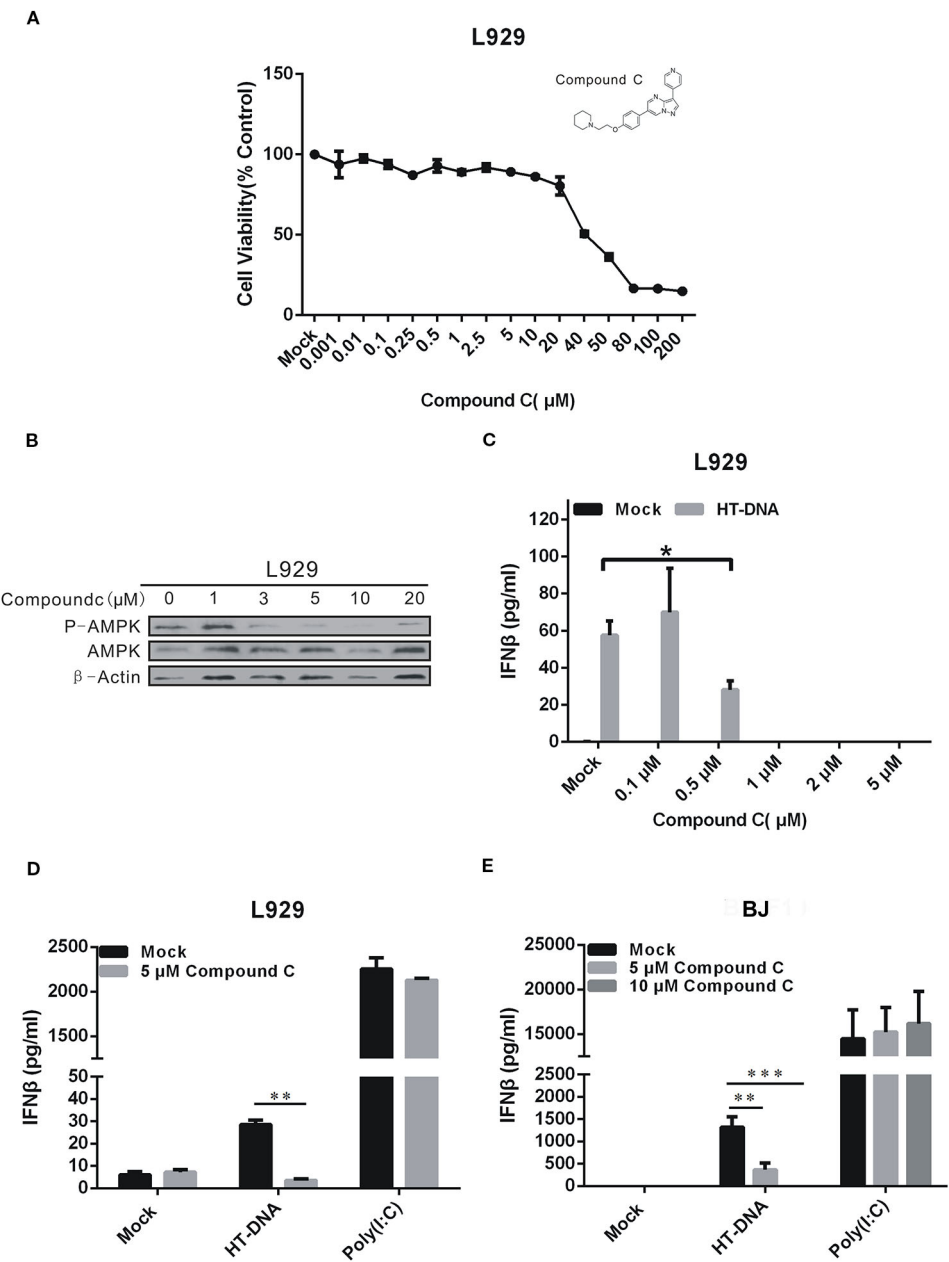
Compound C inhibited type I IFN production in dsDNA signaling. **(A)** L929 cells were treated with the different doses of Compound C for 24 h, and subjected to the CCK-8 assay. **(B)** Compound C inhibited the AMP-activated protein kinase (AMPK) activity as tested by Western blotting. **(C)** Enzyme-Linked Immunosorbent Assay (ELISA) analyses of IFNβ expression in L929 cells. The cells were transfected with HT-DNA after treated with the different doses of Compound C for 1 h. **(D, E)** ELISA analyses of IFNβ expression in L929 and BJ cells. The cells were transfected with HT-DNA and poly (I:C) after treated with indicate doses of Compound C. The experiments were performed at least three times. The statistical analyses were performed by Student’s-*t* test and the data are presented as mean ± SD (n = 3, ^*^ *P* < 0.05, **P < 0.01,***P < 0.001).

Compound C is annotated as an AMPK inhibitor and can affect DNA-induced IFNβ expression (Konno et al., 2013). However, this effect might not involve its anti-AMPK activities due to its multidimensional antikinase activities. We conducted the following studies to address this question. To rule out a possible cytotoxic effect caused by Compound C, we first tested the cell viability by CCK-8 assays (**Figure 1A**). The L929 cell viability was affected by the concentration increment and was dropped by 20% and more than 50% after treatments with 20 and 40 μM Compound C for 24 h, respectively. The inhibitory effect of Compound C on AMPK activities was also tested by examining the AMPK phosphorylation levels. The AMPK phosphorylation was significantly affected by Compound C at the concentration larger than 3 μM (**Figure 1B**). Consistent with the previous findings (Konno et al., 2013), our Enzyme-Linked Immunosorbent Assay (ELISA) data showed that the HT-DNA-induced IFNβ production was significantly suppressed in both L929 and BJ cells by Compound C (**Figures 1C–E**). In contrast, the IFNβ production induced by the RNA surrogate polyinosinic-polycytidylic acid (poly(I:C)) was not significantly affected in these cell types (**Figures 1D–E**) (Konno et al., 2013). To exclude the possible effect due to the high working concentrations of poly(I:C) stimulation, we also titrated down the doses to obtain responses equivalent in magnitude to those elicited by HT-DNA. However, it turned out that Compound C did not inhibit the RNA-mediated IFNβ production at any concentration tested (**Figure S1A**). These data suggest that Compound C specifically affects DNA-mediated IFNβ production in these cell types.

### Compound C Does Not Affect cGAMP-Mediated IFNβ Expression

**Figure 2:**
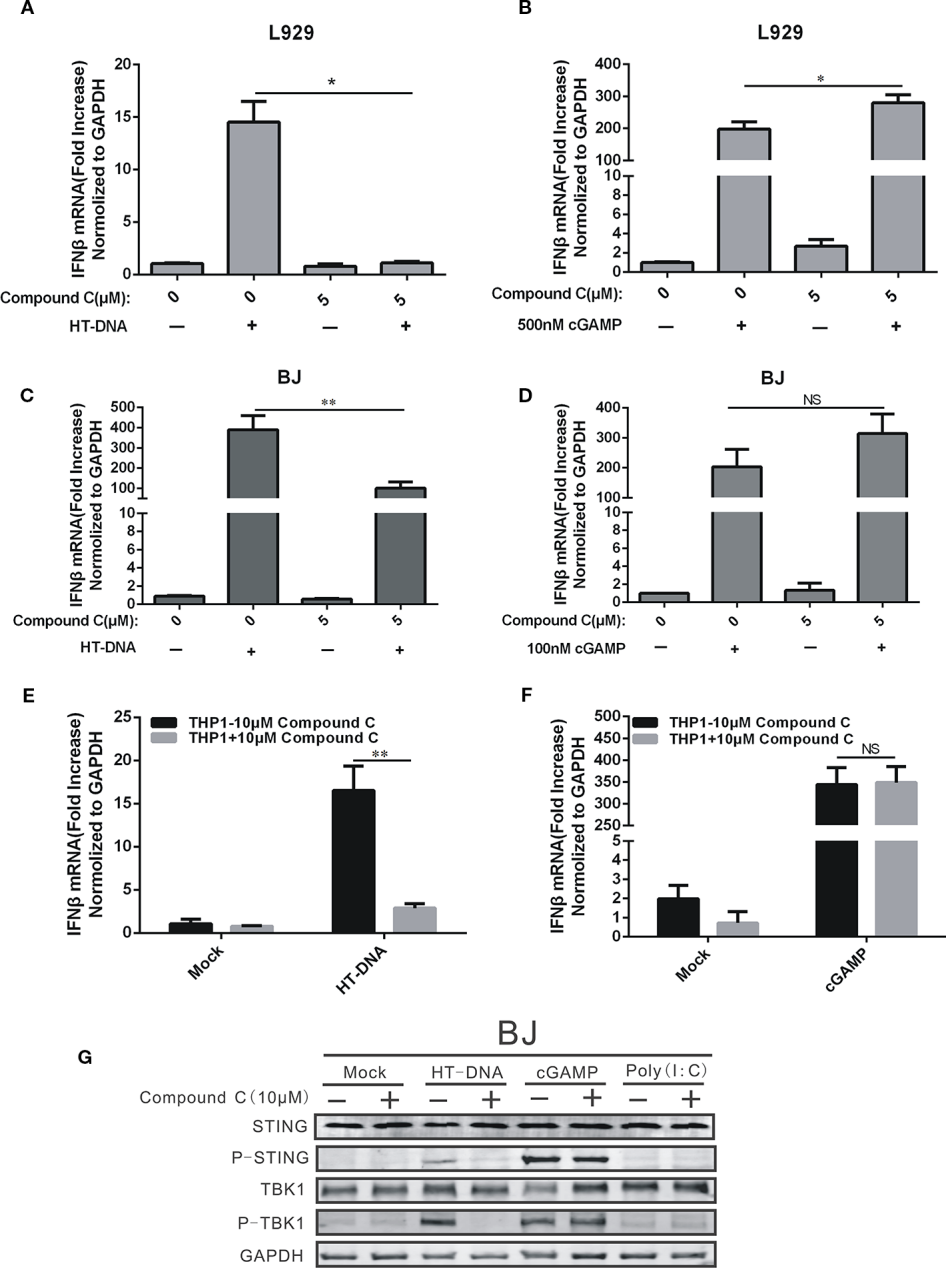
Compound C suppressed the expression of IFNβ by transfected dsDNA, but not cGAMP. **(A, C, E)** RT-qPCR analyses of IFNβ expression in L929 **(A)**, BJ **(C)**, THP1 **(E)** cells. The cells were transfected with HT-DNA after treated with indicated doses of Compound C. **(B, D, F)** RT-qPCR analyses of IFNβ expression in L929 **(B)**, BJ **(D)**, THP1 **(F)** cells. The cells were transfected with poly (I:C) after treated with indicated doses of Compound C for 1 h. **(G)** Western blot analyses of STING, P-STING, TBK1, P-TBK1, and GAPDH expression in BJ cells. The cell was transfected with HT-DNA, cGAMP and poly (I:C) after treated with indicate doses of Compound C. The experiments were performed at least three times. Data in bar graphs are presented as mean ± SD (n = 3−5) with asterisks indicating significant changes (NS Nonsignificance, *P < 0.05, **P < 0.01).

We next examined the Compound C effects on the cGAS-STING pathway which regulates DNA-dependent IFNβ induction. Our RT-qPCR data showed that Compound C significantly suppressed the IFNβ RNA expression-induced by HT-DNA in various cell types including BJ, L929, and THP-1 cells (**Figures 2A, C, E**), but not by cGAMP which was delivered by digitonin permeabilisation. (**Figures 2B, D, F**). Because a recent discovery suggests that the extarcellular cGAMP can be taken up directly by cells *via* the transporter SLC19A1 (Luteijn et al., 2019), we also tested the Commpoun C effect by adding cGAMP to the medium without digitonin permeabilisation. We also did not observe the Compound C effect on cGAMP-mediated IFNβ expression (**Figure 6B**). Next, we examined the Compound C effects on the activities of the regulatory components downstream of cGAMP by Western blotting. Activation of the cGAS-STING pathway is indicated by the phosphorylation of STING and TBK1. In parallel to the above RT-qPCR data, HT-DNA-induced STING and TBK1 protein phosphorylation was significantly inhibited by Compound C (**Figure 2G**) whereas cGAMP and poly(I:C)-induced STING and TBK1 phosphorylation was not affected (**Figure 2G**). We also titrate down the concentrations, but still did not see any effect (**Figure S1B**). Again, these data indicate that Compound C can inhibit cGAS-STING signaling initiated by DNA, and the target site of Compound C is likely to be located upstream of cGAMP.

### Compound C Inhibition on the dsDNA-Dependent Pathway is not Related to its Inhibitory Function on the AMPK Activity

**Figure 3:**
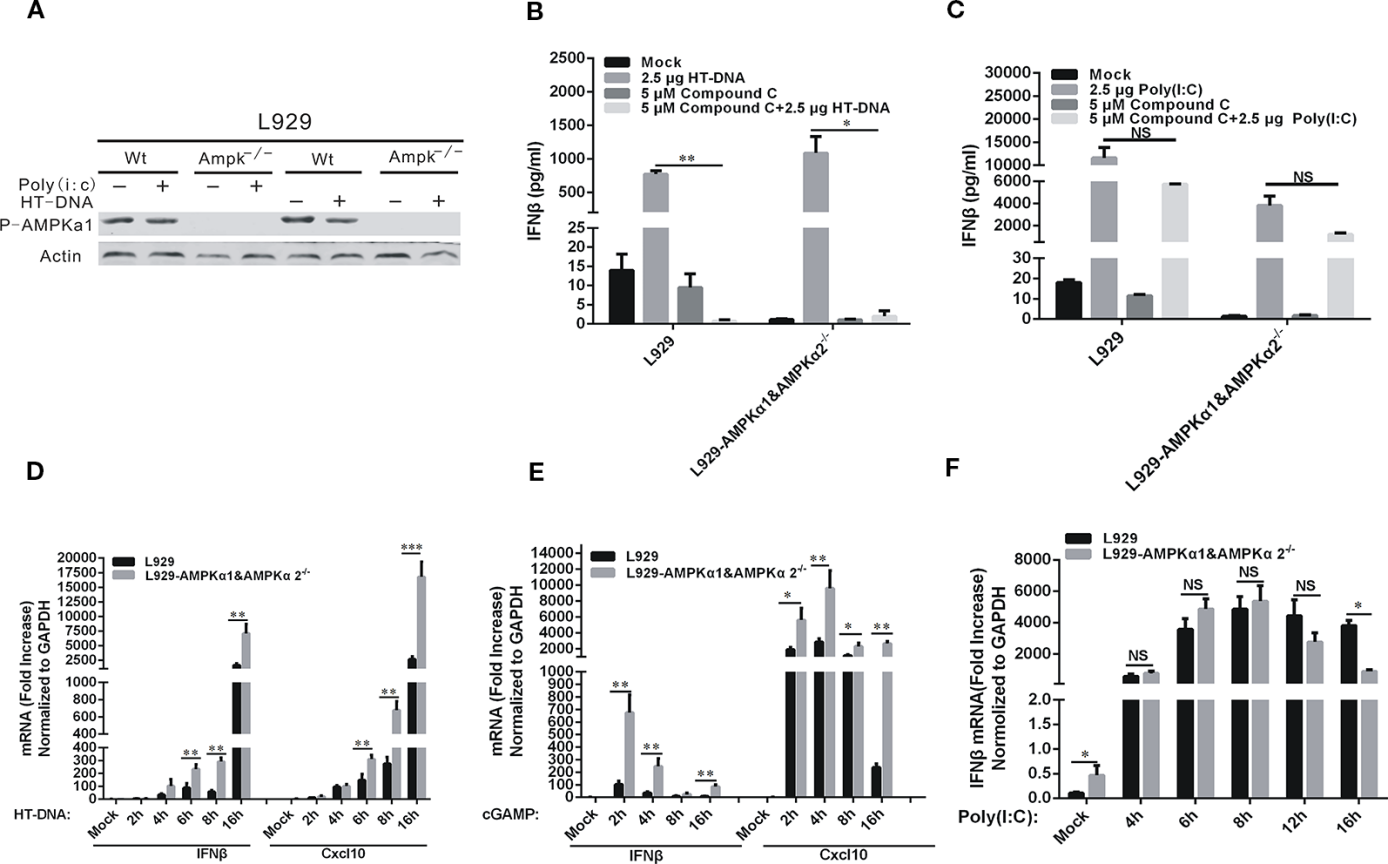
Compound C inhibited the dsDNA-dependent pathway which was not dependent on the activity of AMP-activated protein kinases (AMPKs). **(A)** Western blot analyses of phorsphorylated AMPK in L929 wild-type and L929-AMPK^-/-^ cells. The cells were transfected with HT-DNA and poly (I:C). **(B, C)** RT-qPCR analyses of IFNβ expression in L929 and L929-AMPK^-/-^ cells. The cells were transfected with HT-DNA **(B)** or poly (I:C) **(C)** after treated with indicated dose of Compound C. **(D–F)** RT-qPCR analyses of IFNβ and CXCL10 expression in L929 wild-type and L929-AMPK^-/-^ cells. The cells were transfected HT-DNA **(D)**, cGAMP **(E);** Analyses of IFNβ expression in L929 wild-type and L929-AMPK^-/-^ cells. The cells were transfected with poly (I:C) **(F)**. The experiments were performed at least three times. Data in bar graphs are presented as mean ± SD (n = 5) with asterisks indicating significant changes between the indicated bars (NS, Nonsignificance, ^*^ *P* < 0.05, **P < 0.01,***P < 0.001).

Since Compound C is a potent inhibitor of AMPK, we asked if AMPKα was involved in the inhibitory effects of Compound C on DNA-mediated cGAS-STING signaling. We disabled AMPKα1 and α2 genes in L929 cells by using the CRISPR/Cas 9 gene editing system and established the corresponding gene knockout cell lines, and then studied the effects of Compound C on DNA-induced IFNβ and Cxcl10. Lack of AMPK expression was shown in **Figure 3A** and its expression was not affected by either HT-DNA or poly (I:C) treatments. The induction of IFNβ by HT-DNA did not seem to be affected by AMPK gene knockout and was suppressed by Compound C in both L929 and L929-AMPK^-/-^ cell lines (**Figure 3B**), but Compound C did not affect in poly (I:C)-induced IFNβ (**Figure 3C**). These data suggest that the inhibitory effect of Compound C on DNA-induced IFNβ expression is not dependent on the activity of AMPK (**Figure 3A**). In agreement with the previous studies, AMPK knockout was able to facilitate both HT-DNA and cGAMP-induced IFNβ and CXCL10 mRNA expression (**Figures 3D, E**). However, the deficiency of AMPK has no effect on the dsRNA-dependent pathway (**Figure 3F**). Thereby, the Compound C effects on the dsDNA-dependent pathway are not due to its inhibition of AMPK function.

### The Target of Compound C was in the Upstream of TBK1

**Figure 4:**
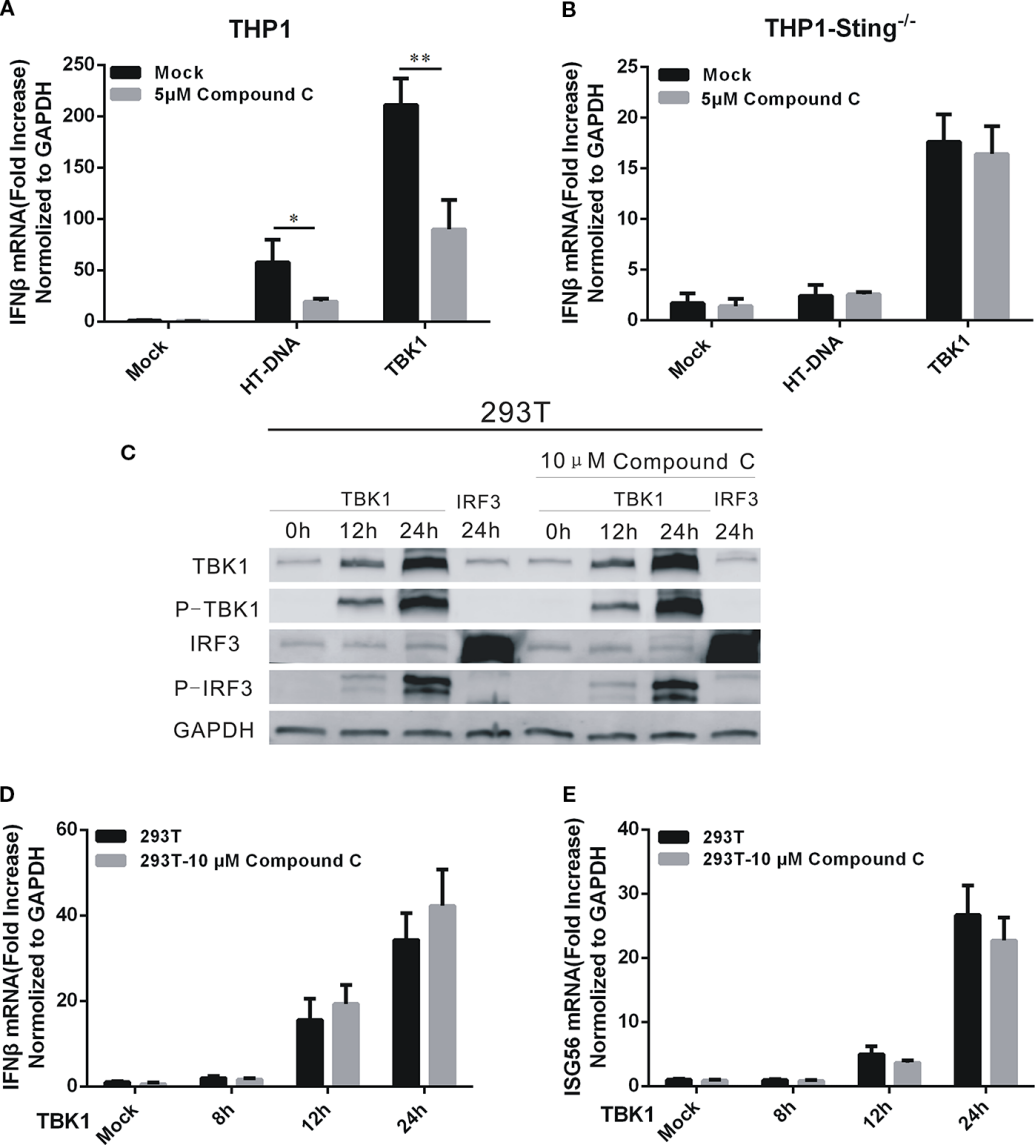
The target of Compound C was in the upstream of TBK1. **(A, B)** RT-qPCR analyses of IFNβ expression levels in THP1 wild-type **(A)** and THP1-STING^-/-^ **(B)** cells. The cells were transfected with HT-DNA and pcDNA 3.1-TBK1-Flag after treated with indicated dose of Compound C. **(C)** Western blot analyses of TBK1, p-TBK1, IRF3, and p-IRF3 expression in 293T cells. The 293T cells were treated with 10 μM Compound C followed by transfection with pcDNA3.1-TBK1-Flag and pcDNA3.1-IRF3-Flag. **(D, E)** RT-qPCR analyses of IFNβ **(D)** and ISG56 **(E)** expression in 293T cells. The 293T cells were treated with 10 μM Compound C followed by transfection with pcDNA3.1-TBK1-Flag for 12 h and 24 h, respectively. The experiments were performed at least three times. The statistical analyses were performed by Student’s-*t* test and the data are presented as mean ± SD (n = 3) with asterisks indicating significant changes (^*^ *P* < 0.05, **P < 0.01).

TBK1 is the downstream protein kinase that drives both DNA- and RNA-mediated production of type I IFN by phosphorylating the transcription factor interferon regulatory factor 3 (IRF3) (Liu et al., 2015). To locate the target of the Compound C action, we performed overexpression of TBK1 to activate the expression of IFNβ in THP1 and THP1-STING^-/-^ cells. As indicated earlier, STING is the immediate upstream adaptor protein that regulates TBK1 activation (Wu et al., 2013). IFNβ was induced by TBK1 overexpression but was dependent on the presence of STING (**Figures 4A, B**). We found that the expression of IFNβ was significantly reduced after 1 h treatment of the THP1 cell line with 5 μM Compound C following HT-DNA and pcDNA3.1-TBK1 transfection, while no effect on the THP1-STING^-/-^ cell line was found (**Figures 4A, B**). So we assumed that the target of Compound C in suppressing dsDNA-dependent IFNβ was in the upstream of TBK1. To further address this issue, we overexpressed TBK1 in 293T cell line in which little cGAS or STING is expressed (**Figure 4C**), and then treated these cells with Compound C. Compound C lost its capability to suppress IRF3 activation when TBK1 was overexpressed (**Figure 4D**). Furthermore, Compound C did not inhibit the expression of IFNβ and ISG56 after TBK1 overexpression (**Figures 4D, E**). Therefore, the target of Compound C on dsDNA-signaling is likely to be located in the upstream of TBK1.

### Compound C Lowers the Level of cGAMP

**Figure 5:**
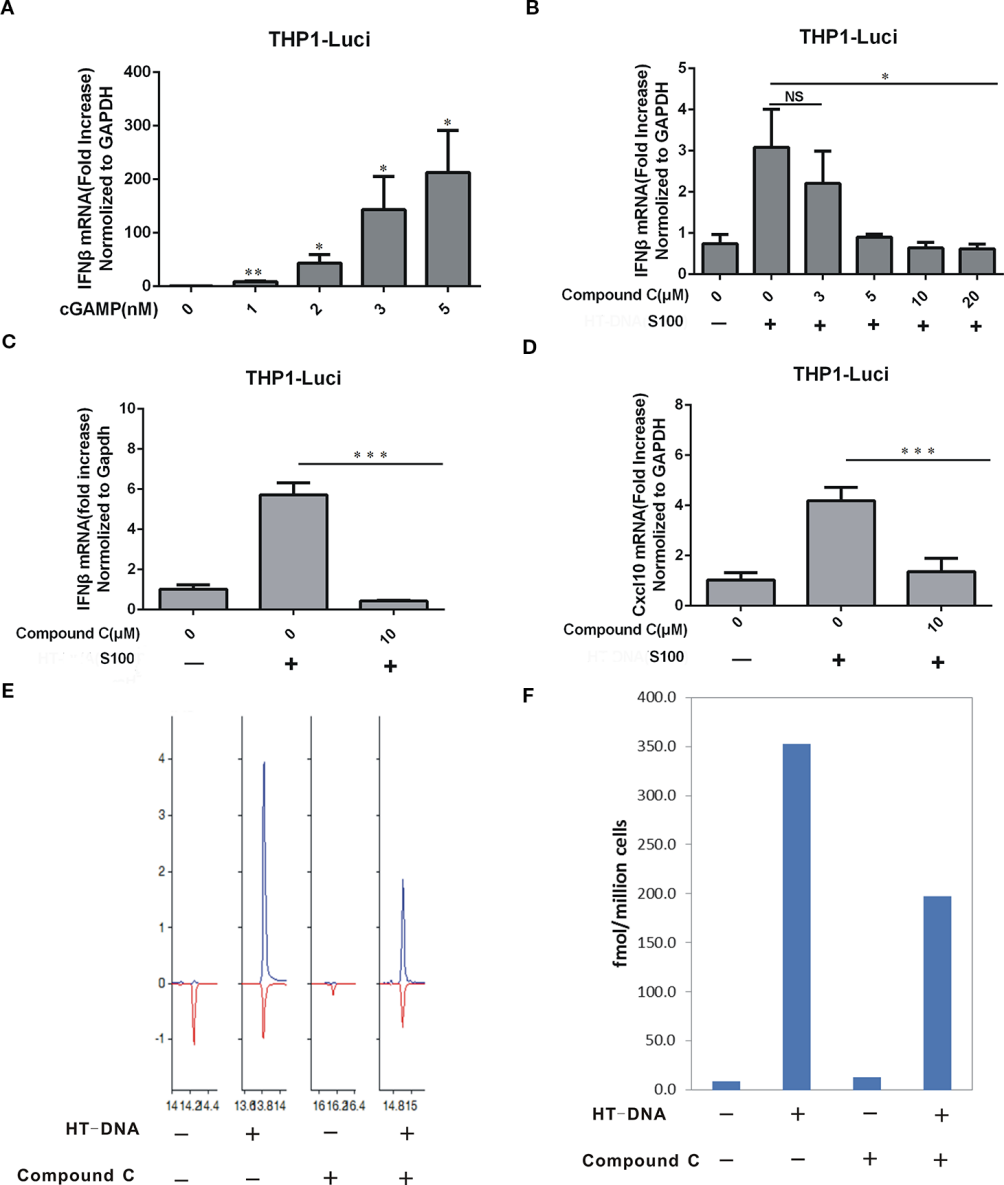
Compound C lowered the cGAMP level. **(A)** RT-qPCR analyses of IFNβ induction in THP1-ISG-liciferase cells. The cells were treated with the increasing amounts of cGAMP. **(B)** RT-qPCR analyses of IFNβ induction in THP1-ISG-liciferase cells. The cells were treated with the S100 fractions which were extracted from the BJ cells transfected with HT-DNA after treated with indicated doses of Compound C for 1h. **(C, D)** RT-qPCR analyses of IFNβ **(C)**, CXCL10 **(D)** expression in THP1-ISG-liciferase cells treated with the S100 fractions which were extracted from the BJ cells transfected with HT-DNA after treated with indicated doses of Compound C for 1h. **(E, F)** LC-MS analyses of the content of cGAMP in the S100 fractions which were extracted from the BJ cells transfected with HT-DNA after treated with 10 μM Compound C. The experiments were performed at least three times. Data in bar graphs are presented as mean ± SD (n = 3−5) with asterisks indicating significant changes (NS, Nonsignnificant, ^*^ *P* < 0.05, **P < 0.01,***P < 0.001).

To evaluate the Compound C effect on the cGAMP level, we treated BJ cells with combined HT-DNA and Compound C (at various concentrations) and the cytoplasmic extract (S100), which supposed to contain the endogenous cGAMP, was isolated and used to treat THP-Luci cells to induce the IFNβ production. Commercially purchased cGAMP was also used as control (**Figure 5A**). The RT-qPCR data show that the mRNA levels of IFNβ induced by the S100-fraction were largely reduced in the THP1-luci-ISG cell line by Compound C at the concentrations higher than 5 μM, suggesting that Compound C strongly lowered the cGAMP level (**Figures 5B, C**). In addition to IFNβ, the mRNA expression of CXCL10 was also inhibited in the presence of Compound C (**Figure 5D**). To more directly assess the level of cGAMP, we measured the cGAMP content in the S100 extracts collected from BJ cells treated with HT-DNA or combined treated with Compound C by LC-MS. The data showed that Compound C significantly lowered HT-DNA-induced cGAMP level (**Figures 5E, F**). These data suggest that Compound C is able to lower the cGAMP level.

### Compound C Does not Affect the cGAS Enzymatic Activity *In Vitro*

**Figure 6:**
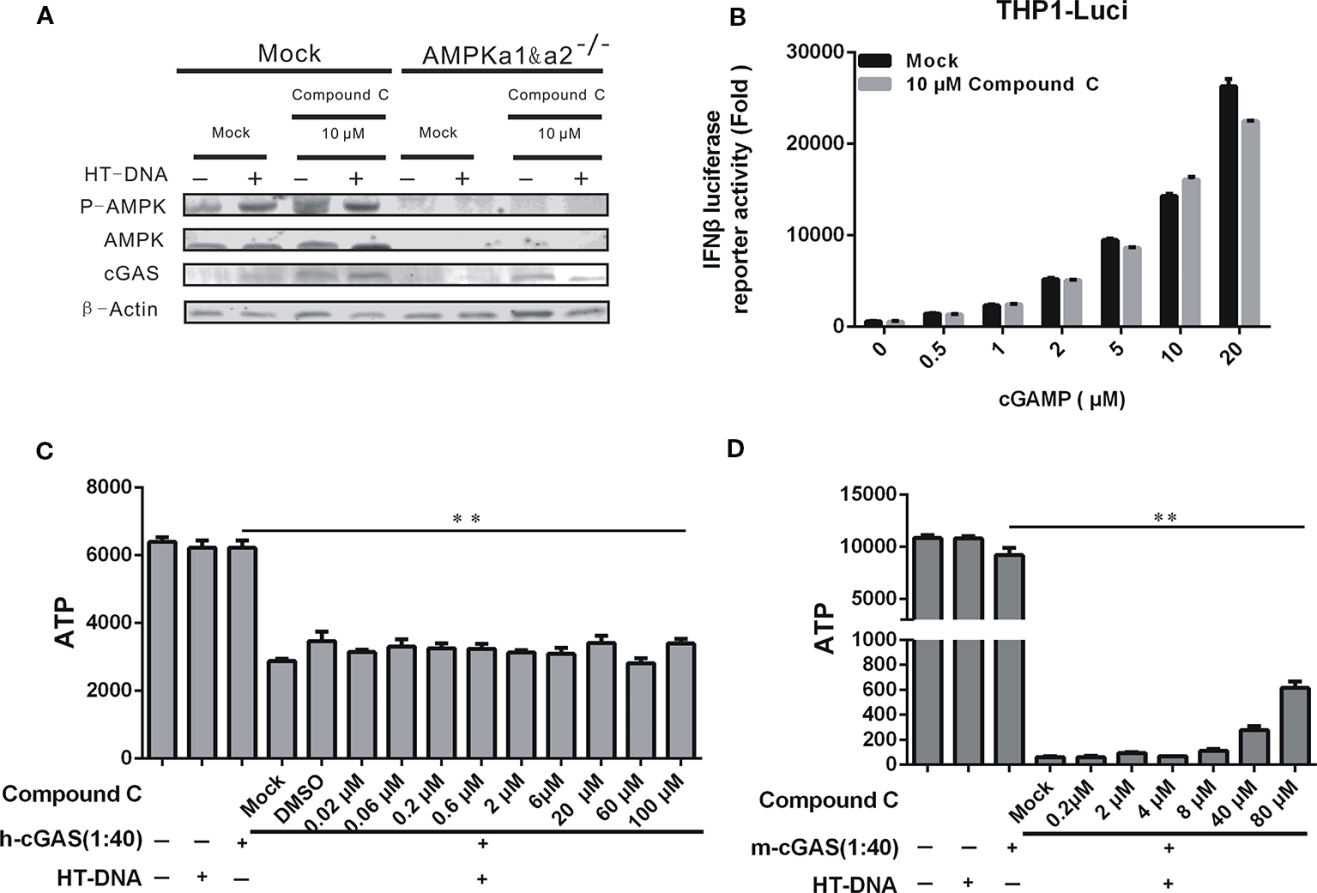
The inhibitory effect of Compound C on cGAS-STING signaling was not dependent on the cGAS enzymatic activity. **(A)** Western blot analyses of p-AMPK, AMPK, and cGAS expression in L929 wild-type and L929-AMPK^-/-^ cells. The cells were transfected with HT-DNA and poly (I:C) after treated with indicated doses of Compound C. **(B)** Enzyme-Linked Immunosorbent Assay (ELISA) analyses of IFNβ expression in THP1-Luci cells following stimulation by cGAMP at the different concentrations for 16 h with or without 5 μM Compound C treatments. **(C, D)** The cGAS enzyme activity was evaluated by the (ATP consumption. The experiments were performed at least three times. Data in bar graphs are presented as mean ± SD (n = 3−5) with asterisks indicating significant changes (**P < 0.01).

Since the cGAMP level could be affected by cGAS-mediated synthesis, we tested the cGAS enzymatic activity. First, the expression levels of cGAS protein in L929 and L929-AMPK-KO cells were analyzed by Western blotting, and the data showed that the expression of cGAS protein was not affected by Compound C in both the wild type and AMPK gene knockout L929 cells (**Figure 6A**). We next asked if Compound C could affect the cGAS enzymatic activity *in vitro*. We examined ATP consumption during the cGAMP synthesis *in vitro*, but did not see any effect (**Figures 6C, D**). Therefore, the suppression of Compound C on DNA-mediated cGAS-STING signaling activation is not affected by the cGAS enzymatic activity. We also aksed if Compound C could affect cGAMP transportation, We treated THP1-Luci cells by 5 μM Compound C along with cGAMP at the high concentrations (at which cGAMP is supposed to be transported into cells (Luteijn et al., 2019) without digitonin permeabilisation for 16 h, then cGAMP transshipment was examined by Multiscan Spectrum). In fact, we did not observe the Compound C effect on cGAMP transshipment (**Figure 6B**). Thus, Compound C does not affect cGAS enzymatic activity.

### Compound C Ameliorates Autoimmune Phenotypes Induced by Loss of Trex1 Gene *In Vitro* Cells

**Figure 7:**
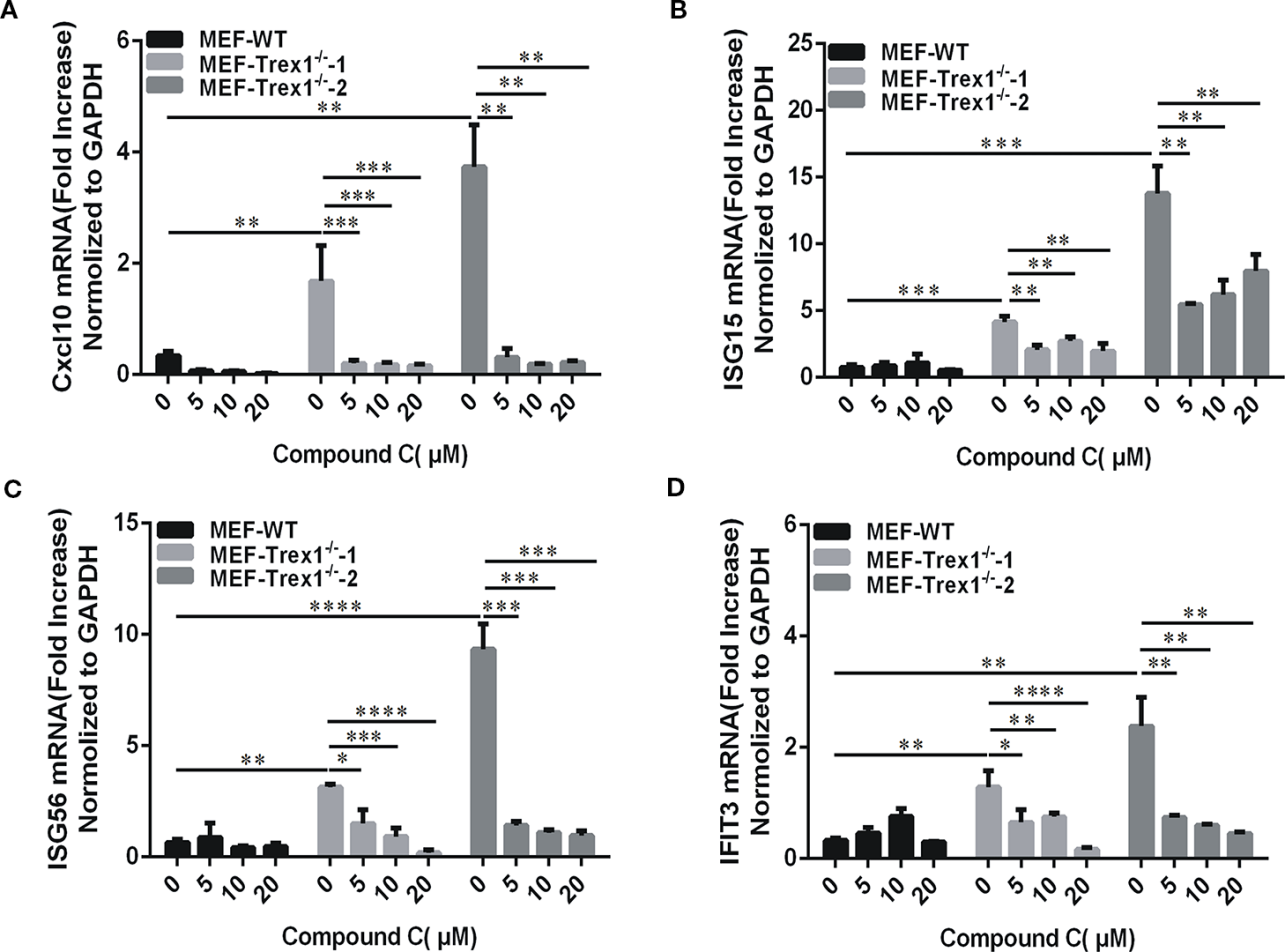
Compound C is active in cells loss of Trex1 gene. **(A–D)** RT-qPCR analyses of CXCL10 **(A)**, ISG15 **(B)**, ISG56**(C)**, and IFIT3 **(D)** expression in mouse embryonic fibroblast (MEF) wild-type and MEF-Trex1**^-/-^** cells. The cells were treated with indicated doses of Compound C for 3 h. The experiments were performed at least three times. Data in bar graphs are presented as mean ± SD (n = 3) with asterisks indicating significant changes (^*^P < 0.05, ^**^P < 0.01, ^***^P < 0.001, ^****^P < 0.0001).

TREX1 is a major cytoplasmic exonuclease that degrades dsDNA and ssDNA (Yun-Gui et al., 2007; Nan et al., 2010). The TREX1 gene Mutations have been linked to the autoimmune diseases including Aicardi-Goutières syndrome (AGS) and systemic lupus erythematosus (SLE) (Crow et al., 2006). The autoimmune phenotypes derived from the Trex1 gene mutations in mice can be ameliorated by cGAS or STING gene knockout (Gao et al., 2015; Xiao et al., 2019). Since Compound C displays an inhibitory effect on the cGAS-STING-mediated pathway, we further determined its potential beneficial effects against autoimmune phenotypes in the Trex1 mutant cells. We isolated the mouse embryonic fibroblasts (MEFs) from the Trex1 knockout mice. Since the half-life of IFNβ is short and the detection time is not well established in MEF-Trex1^-/-^, we examined downstream ISGs instead, but not IFNβ itself, to verify the Compound C inhibitory effect on cGAS-STING activation caused by Trex1 deletion. These cells displayed the elevated levels of CXCL10, ISG15, ISG56, and IFIT3 and their expression was significantly suppressed by Compound C (**Figures 7A–D**), suggesting the effectiveness of Compound C in inhibiting intrinsic DNA-dependent, constitutively activated type I IFN expression in the cells deficient in TREX1. Therefore, Compound C may be used as a molecular scaffold for the development of autoimmune therapy in the future.

## Discussion

Despite the cellular immune response to dsDNA plays an indispensable role in pathogen defense, abnormal response to dsDNA has been shown to be an important factor in the etiology of hyperinflammatory or autoimmune disorders, such as SLE (Pisetsky, 2016) and AGS (Crow et al., 2006) or Chilblain lupus (Rice et al., 2007). But so far, there is no effective therapy for these diseases. Compound C is a small molecule compound widely used as an inhibitor of AMPKs. In the present study, we find that Compound C can be used as an inhibitor of the DNA-dependent cGAS-STING pathway. Our data demonstrate that after the HT-DNA transfection, the expression of IFNβ was significantly inhibited in human or mouse cells treated with Compound C. We also tested if Compound C was able to inhibit the activation of RNA-sensing pathway by using poly(I:C) treatment. However, we did not see the consistent effect and the Compound C effects on RNA-mediated immune responses seemed to be cell-type dependent. Particularly, we observed Compound C-mediated inhibition of the IFNβ mRNA expression induced by poly(I:C) only in the cells that express little or no cGAS and STING (**Figure S2** and **Figure S3**), which needs to be further clarified in the future.

In this study, since Compound C did not significantly affect cGAMP-induced INFβ production and it may work in the upstream of cGAMP. Indeed, in the experiments by testing the Compound C effects following TBK1 stimulation in the cells lacking cGAS-STING, we further demonstrate that the target of compound C is in the upstream of TBK1. We next tested if Compound C could directly affect the function of cGAS. However, Compound C did not decrease either the protein level of cGAS in the cells tested or the ATP consumption in *in vitro* cGAMP synthesis assay (**Figure 6**). In addition, MicroScale Thermophoresis (MST) experiments showed the binding affinity (Kd) of Compound C to the cGAS was 96.6 μM, but we were unable to solve the crystal structure of Compound C in complex with cGAS and dsDNA (data not shown). Therefore, Compound C does not directly bind to the cGAS active sites. Therefore, Compound C may inhibit the cGAS-mediated activites by inhibiting a certain upstream gene, a similar role as epigallocatechin gallate (EGCG). For example, EGCG is an inhibitor for GTPase-activating protein SH3 domain-binding protein 1 (G3BP1), which is critical for the cGAS activation since it promotes the G3BP1-cGAS complex formation and enhances DNA binding of cGAS (Liu et al., 2018). Alternatively, Compound C may reduce cGAMP accumulation by facilitating cGAMP degradation. Indeed, our intracellular extraction of cGAMP and LC/MS experiments showed that the cells treated with Compound C caused a significant reduction of cGAMP after stimulation with HT-DNA (**Figure 5**). Interestingly, a recent study shows that Ecto-nucleotide pyrophosphatase phosphodiesterase 1 (ENPP1), preferentially hydrolyzes 2′3′-cGAMP, but not 3′3′-cGAMP, thereby negatively regulates the cGAS-STING pathway (Wang et al., 2018a). This preferential degradation is due to its binding to the ENPP1 active site in a conformation suitable for catalysis (Kato et al., 2018). Thus, it needs to be further clarified if Compound C can affect cGAMP degradation by modulating ENPP1 activities.

It has been reported that cGAS is essential in activating the AMPK/ULK1 to suppress STING, which is responsible for triggering the dephosphorylation of AMPK T172 and activation of ULK1, so as to phosphorylate STING on S366 to impede its activity (Konno et al., 2013). Thus, the phosphorylation of AMPK may inhibit the cGAS-STING pathway. However, loss of AMPK function does not affect the IFNβ mRNA expression induced by HT-DNA or cGAMP in the present study since Compound C-mediated inhibition of the IFNβ mRNA expression is also found in the L929-AMPK^-/-^ cell line, suggesting that the AMPK may not be responsible for Compound C-mediated inhibition of the IFNβ mRNA expression, and some other targets might be involved.

In addition to AMPKs, ALKs which are the major kinases involved in BMP-mediated signaling is another major kinase family that can be inhibited by Compound C. Thus, ALKs may be a possible candidate that is involved in Compound C-mediated inhibition. To test this hypothesis, we generated the ALKs knock-out cells and we found that the production of IFNβ was inhibited by the loss of ALKs when these gene knock-out cells were transfected with HT-DNA (data not shown). We currently work on this hypothesis but the data will not be included here.

Apart from AMPK and BMP signaling, Compound C has been shown to exert various ‘‘off-target’’ biological effects, such as inhibiting vascular endothelial growth factor type II receptor and inhibiting hypoxia-inducible factor-1 activation (Fraley et al., 2002; Emerling et al., 2007). By using the kinase inhibition profiling panel, Compound C has been found to inhibit a number of other kinases with similar or greater potencies (Bain et al., 2007). Therefore, we cannot exclude the other protein kinase pathways that may contribute to Compound C-mediated inhibition of the immune responses.

Due to the deficiency of Trex1^−/−^ and DNaseII^−/−^ in mice, impaired aberrant DNA clearance causes severe autoimmune phenotypes, including inappropriate activation, continuous production of type I IFN and a high lethality (Napirei et al., 2000; Morita et al., 2004; Okabe et al., 2005; Yoshida et al., 2005). These autoimmune phenotypes can be rescued by genetic ablation of STING, IRF3 or the type I IFN receptor (Kawane et al., 2006; Stetson et al., 2008; Ahn et al., 2012; Gall et al., 2012; Ablasser et al., 2014; Gao et al., 2015; Gray et al., 2015). The abnormal activation of cGAS in autoimmune diseases caused by incomplete self-DNA clearance has also been demonstrated in *Trex1* ^−/−^ and DNaseII^−/−^ mice. Activation of cGAS-STING results in an autoimmune phenotype in the mice with Trex1 and DNaseII deletions. Deletion of cGAS or STING improves the survival and autoimmune phenotypes such as elevated ISG expression, production of autoantibodies of Trex1- or DNaseII-deficient mice by preventing cGAMP accumulation and ISGs expression (Gao et al., 2015; Motani et al., 2015). Therefore, keeping cGAS-STING function in line could be a potential therapeutic strategy for the diseases caused by reduced TREX1 activity, such as lupus. In our study, we showed the Compound C effects in autoimmune inhibition by using MEF-Trex1^-/-^ cell lines. Therefore, Compound C may have potential therapeutic values for being used as a potent inhibitor of dsDNA-induced autoimmune activation, such as treating immune diseases caused by Trex1 deletion.

In summary, cGAS is an essential protein for the innate immune response to cytosolic DNAs and has been considered as a potential valuable target for the therapeutic development to improve the treatment of human autoimmune disorders. Compound C showed potent and selective inhibition of dsDNA-dependent pathway, which may shed light on developing the immunomodulatory therapeutic agents in treating cGAS-related human disorders.

## Data Availability Statement

All datasets generated for this study are included in the article/**Supplementary Material**.

## Ethics Statement

The animal experiments were performed under the Guide for the Care and Use of Laboratory Animals approved by Fujian Provincial Office for Managing Laboratory Animals and were overseen by the Fujian Normal University Animal Care and Use Committee.

## Author Contributions

Conceptualization: QC. Methodology: JL, XL, ST, XZ. Validation: JL, ST, QL, SH. Formal analysis: JL, HW. Investigation: JL, HW, SC. Data curation: JL. Writing (original draft preparation): QC, JL, ST. Writing (review and editing): QC, JL, ST. Visualization: JL, XL. Supervision: QC. Project administration: QC. Funding acquisition: QC.

## Funding

This research was supported by the Natural Science Foundation of the Fujian Province, China (Grant No. 2017J01621) and the Innovative Research Team Program II of Fujian Normal University in China (IRTL1703).

## Conflict of Interest

The authors declare that the research was conducted in the absence of any commercial or financial relationships that could be construed as a potential conflict of interest.
