# Supplementary material for: Compound C Reducing Interferon Expression by Inhibiting cGAMP Accumulation
Source: Front Pharmacol. 2020 Feb 28;11:88. doi: 10.3389/fphar.2020.00088 (PMC7059800; doi:10.3389/fphar.2020.00088)
Supplement: Supplementary file 1 [file DataSheet_1.docx]

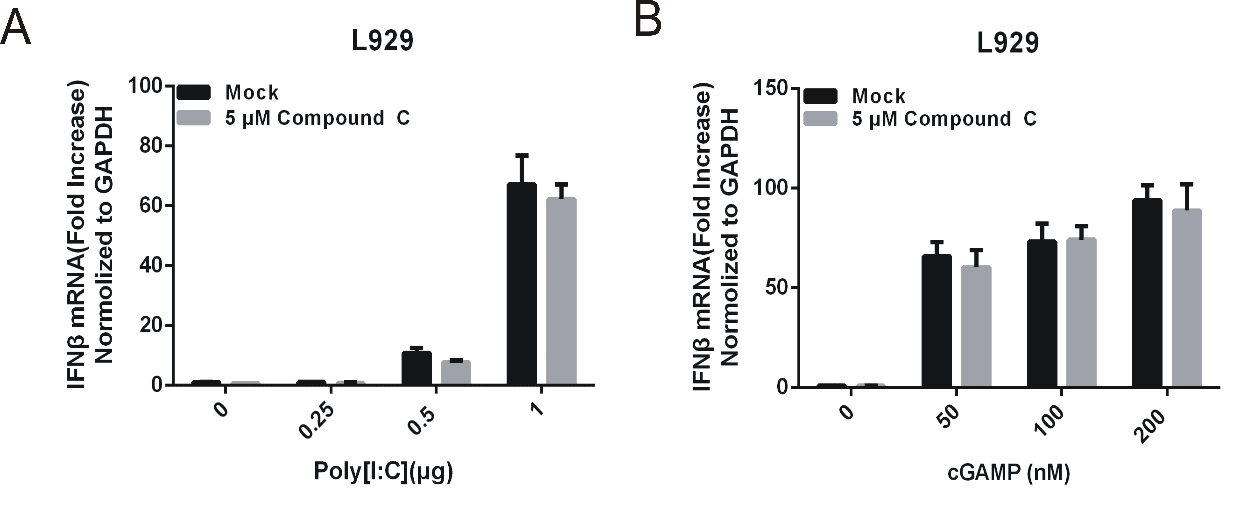


Figure S1. RT-qPCR analysis of IFNβ expression levels in L929 cells which were transfected with poly (I:C) **(A)** or cGAMP **(B)** at different concentrations after treated with 5 μM Compound C for 1 h .

**
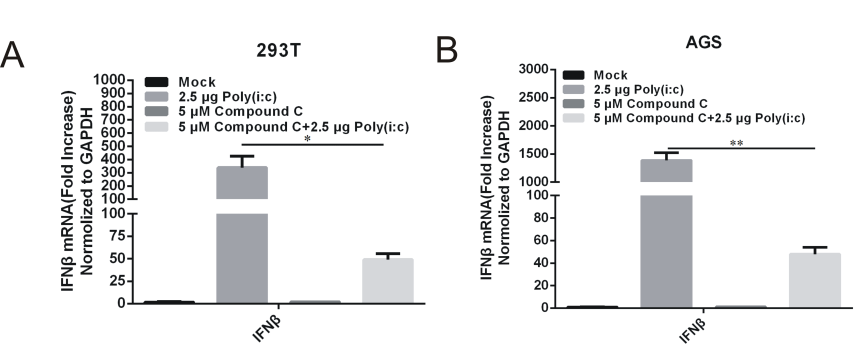
**

Figure S2. Compound C suppressed dsRNA-dependent type I interferon induction in AGS and 293T cells which none or very little cGAS or STING is expressed. RT-qPCR analysis of IFNβ expression levels in 293T **(A)** and AGS **(B)** cells which were transfected with poly(I:C) after treated with 5 μM Compound C for 1 h . The statistical analyses were performed by Student’s-*t* test and the data are presented as mean ± SD (n = 3, **P* < 0.05, ***P* < 0.01).

**
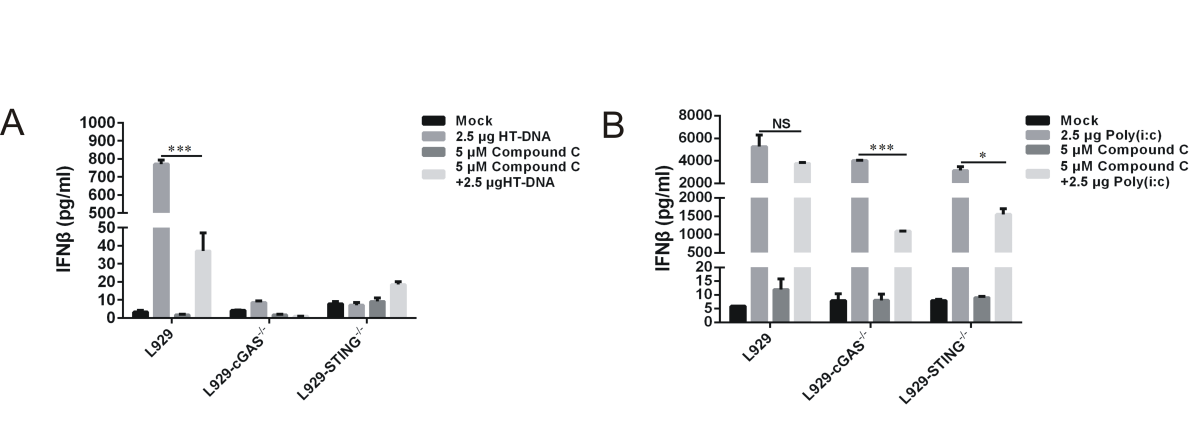
**

Figure S3. Compound C suppressed dsRNA-dependent type I IFN induction in the cells lack of cGAS or STING expression. ELISA analysis of IFNβ expression in L929, L929-cGAS^-/-^ and L929-STING^-/-^ cells were exposed to HT-DNA **(A)** and poly (I:C) **(B)** after treated with 5 μM Compound C for 1 h. Data in bar graphs are presented as mean ± SEM (n = 3) with asterisks indicating significant changes between the indicated bars (NS Nonsignificance, ^*^*P* < 0.05, ^***^*P* < 0.001).

| Table S1. List of forward and reverse primers used in qRT-PCR | | |
| --- | --- | --- |
| Target | Forward (F) | Reverse (R) |
| M-GAPDH | CCAGAGCTGAACGGGAAGCTCAC | CCATGTAGGCCATGAGGTCCACC |
| M-IFNβ | TGGGTGGAATGAGACTATTGTTG | CTCCCACGTCAATCTTTCCTC |
| M-CXCL10 | GCCGTCATTTTCTGCCTCA | CGTCCTTGCGAGAGGGATC |
| M-ISG56 | ATCGCGTAGACAAAGCTCTTC | GTTTCGGGATGTCCTCAGTTG |
| M-ISG15 | TCTTTCTGACGCAGACTGTAG | GGGGCTTTAGGCCATACTCC |
| M-IFIT3 | CCTACATAAAGCACCTAGATGGC | ATGTGATAGTAGATCCAGGCGT |
| H-GAPDH | GGAGCGAGATCCCTCCAAAAT | GGCTGTTGTCATACTTCTCATGG |
| H-IFNβ | ATGACCAACAAGTGTCTCCTCC | GGAATCCAAGCAAGTTGTAGCTC |
| H-CXCL10 | GTGGCATTCAAGGAGTACCTC | TGATGGCCTTCGATTCTGGATT |
| H-ISG56 | AGAAGCAGGCAATCACAGAAAA | CTGAAACCGACCATAGTGGAAAT |
